# Validating a GPS-based approach to detect health facility visits against maternal response to prompted recall survey

**DOI:** 10.7189/jogh.10.010602

**Published:** 2020-06

**Authors:** Andrew Marsh, Siddhivinayak Hirve, Pallavi Lele, Uddhavi Chavan, Tathagata Bhattacharjee, Harish Nair, Harry Campbell, Sanjay Juvekar

**Affiliations:** 1 Institute for International Programs, Johns Hopkins University Bloomberg School of Public Health, Baltimore, Maryland, USA; 2 KEM Hospital Research Centre, Sardar Moodliar Road, Rasta Peth, Pune, India; 3 INDEPTH Network, 40 Mensah Wood Street, East Legon, Accra, Ghana; 4 Usher Institute of Population Health Sciences and Informatics, University of Edinburgh, Edinburgh, UK

## Abstract

**Introduction:**

Common approaches to measure health behaviors rely on participant responses and are subject to bias. Technology-based alternatives, particularly using GPS, address these biases while opening new channels for research. This study describes the development and implementation of a GPS-based approach to detect health facility visits in rural Pune district, India.

**Methods:**

Participants were mothers of under-five year old children within the Vadu Demographic Surveillance area. Participants received GPS-enabled smartphones pre-installed with a location-aware application to continuously record and transmit participant location data to a central server. Data were analyzed to identify health facility visits according to a parameter-based approach, optimal thresholds of which were calibrated through a simulation exercise. Lists of GPS-detected health facility visits were generated at each of six follow-up home visits and reviewed with participants through prompted recall survey, confirming visits which were correctly identified. Detected visits were analyzed using logistic regression to explore factors associated with the identification of false positive GPS-detected visits.

**Results:**

We enrolled 200 participants and completed 1098 follow-up visits over the six-month study period. Prompted recall surveys were completed for 694 follow-up visits with one or more GPS-detected health facility visits. While the approach performed well during calibration (positive predictive value (PPV) 78%), performance was poor when applied to participant data. Only 440 of 22 251 detected visits were confirmed (PPV 2%). False positives increased as participants spent more time in areas of high health facility density (odds ratio (OR) = 2.29, 95% confidence interval (CI) = 1.62-3.25). Visits detected at facilities other than hospitals and clinics were also more likely to be false positives (OR = 2.78, 95% CI = 1.65-4.67) as were visits detected to facilities nearby participant homes, with the likelihood decreasing as distance increased (OR = 0.89, 95% CI = 0.82-0.97). Visit duration was not associated with confirmation status.

**Conclusions:**

The optimal parameter combination for health facility visits simulated by field workers substantially overestimated health visits from participant GPS data. This study provides useful insights into the challenges in detecting health facility visits where providers are numerous, highly clustered within urban centers and located near residential areas of the population which they serve.

An estimated 5.9 million children under five die each year globally with pneumonia, malaria, and diarrhea among the leading causes [1]. Proven preventative and curative interventions exist to reduce mortality and morbidity from these causes yet their impact is limited by poor access to health care [2]. Data on various household health behaviors, including care-seeking for childhood illness, are commonly collected through large-scale household surveys, such as the Demographic and Health Survey and the Multiple Indicator Cluster Survey [3,4]. These data typically rely on maternal self-report, the validity of which is subject to various biases [5]. Technology-based approaches to collect information on participant behavior, especially those using Global Positioning System (GPS) sensors, provide an alternative to survey-based approaches, minimizing the biases inherent in those traditional approaches while broadening the range of topics which may be explored [6]. Many such approaches have been applied to study health behaviors though their validity is not always explored and may depend both on the behavior being considered and the context within which it is being assessed.

GPS has been increasingly applied within the field of health research. Depending on satellite visibility and location, GPS receivers are capable of continuously determining their location with a precision of several meters [7]. Among the many applications of GPS are the study of disease exposure and transmission [8-10], environmental exposure [11,12], social interaction and exclusion [13,14], mobility-related illness outcomes [15], and physical activity [16]. While GPS has traditionally been measured through dedicated devices, GPS receivers have become a common feature in smartphones and have been shown to produce comparable data to traditional devices [11,17,18]. Furthermore, GPS-enabled smartphones supplement traditional approaches with data from the cellular network to improve the speed with which location data are obtained [18].

Raw participant GPS records include latitude, longitude, accuracy, and the time at which the coordinate was recorded. These records alone are insufficient to draw inferences about participant behaviors and require post-processing to extract meaningful data on periods of movement, travel mode, and significant locations visited [6]. Geofencing and cluster-detection approaches are two commonly used approaches to infer visited locations from participant GPS data [19-21]. Geofencing requires that the researcher knows the location for each feature of interest (eg, health facility) where visits are to be detected. A boundary is then defined around each feature, which may take the form of the building’s footprint or may be a radius about a specified point of interest. Participant GPS data are then examined for sequential points located within the specified boundary, classifying those points as a visit when the number of points or the duration between the first and last point meet some predefined threshold. Several cluster-based approaches exist for visit detection with substantial variability in their methods [22-25] One example, ST-DBSCAN, identifies clusters according to three parameters: a minimum number of points, and a maximum spatial and non-spatial (eg, time) distance that these points are located from one another [26]. Cluster-based approaches are more flexible than geofencing approaches in that they do not require any knowledge of where features are located, however they tend to require larger data sets and consequently increased computing power.

Both approaches require user-specified parameters, optimal values for which vary with study aim, setting, and the mechanics of the specific approach being applied. Overly restrictive parameters will fail to detect true visits while overly permissive parameters will falsely classify non-visits as visits. Studies identifying visited locations within participant movement data commonly apply duration thresholds of 20 to 30 minutes [14,20,27,28], though duration values as low as 5 minutes have been demonstrated to reliably detect activity locations [29]. The selection of a duration threshold relates to the behavior under investigation. In contrast to studies with the objective of identifying locations visited for any purpose, a study specifically identifying hospitalizations set a correspondingly higher duration threshold of 4 hours [19]. There is less convergence around an optimal distance threshold with even those studies using similar duration parameters applying distance values ranging from 10 to 200 m [14,28]. While several studies evaluate the performance of their approach after the fact, the specification of parameter values is typically based on *a priori* assumptions of optimal thresholds. A notable exception to this, Theirry et al. evaluated the performance of six parameter sets on 750 simulated trajectories to determine the optimal values [29]. Such exercises require a calibrating data set where the true value is known for each record, which may not be feasible in all contexts. Given the sensitivity of visit detection approaches to parameter specification, the value of undertaking such a calibration exercise should not be overlooked.

The present study was conducted within the context of a larger study comparing maternally-reported care-seeking behavior with measures of care-seeking behavior derived from participant GPS data [30]. A two-step approach was used to detect health facility visits wherein: 1) optimal parameter values were first identified through a calibration exercise involving researcher simulated health facility visits and 2) these parameter thresholds were applied to the prospective detection of participant health facility visits, the performance for which was assessed through monthly prompted recall surveys. This paper aims to describe the development and implementation of a GPS-based visit detection approach, evaluate its performance for correctly identifying health facility visits, and explore factors associated with maximizing its performance.

## METHODS

### Study site and health facility census

**Figure 1:**
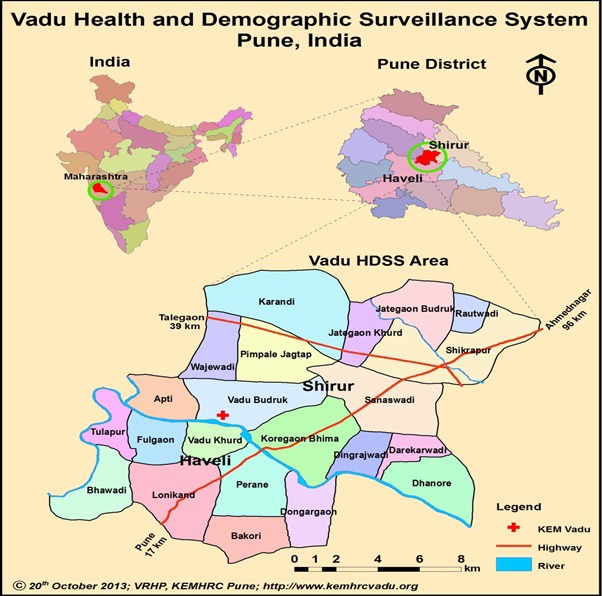
Map of Vadu Health and Demographic Surveillance System (HDSS) within Maharashtra state and Pune district. Note: HDSS – Health and Demographic Surveillance System, km – kilometre. Reproduced with permission from Ingole et al. (2015) [31].

We conducted a prospective cohort study in the 22 villages of the Vadu Health and Demographic Surveillance System, located in rural Pune district, Maharashtra state, India (**Figure 1**). Prior to study initiation we conducted a census of all locations where a mother might seek care for childhood illness, including private, public, formal, and informal providers. Field workers classified each provider by type, obtaining the location coordinates for each provider using a handheld Garmin e-Trex GPS device (Garmin Ltd, Olathe, KS, USA).

### Participant enrollment and follow up

Participants were mothers ages 15-49 with at least one child under five years of age, randomly sampled from the population register of the Vadu HDSS and enrolled during field worker home visits. Consenting participants were randomly assigned to either the primary study group (“phone group”), cross-sectional comparison group, or longitudinal comparison group. Participants in the phone group were provided with a GPS-enabled smartphone and visited monthly during the six-month study period. Comparison group participants were not provided with phones and were visited either once (cross-sectional group) or monthly (longitudinal group) over the study duration. These comparison groups were included to evaluate potential biases in reported care-seeking behavior resulting from providing participants with smartphones and following them up over time, the results of which are described elsewhere [32]. Given the aims of this analysis, the focus is restricted exclusively to participants in the phone group.

Phone group participants were provided with a Sony Xperia E4 Dual SIM phone (Sony Corporation, Tokyo, Japan), a protective case, and a SIM card allowing unlimited mobile data and in-network communication. The costs for these items and any required maintenance during the study period were covered by the research budget. Additional costs, such as those incurred by purchasing paid applications or making in-application purchases, were the responsibility of participants. Participants were asked to charge the phone daily, keep it on during the day, and maintain certain phone settings according to study specifications (eg, mobile data on, location settings set to “high accuracy”). We asked that participants carry the phone whenever seeking care. Otherwise, participants were encouraged to use the phone as if it were their own. Participants could use the secondary SIM slot for their own SIM card, though this action was not necessary for the phone to function. Field workers assisted with any participant queries regarding device use and study instructions when distributing the phones as well as at support visits conducted three and 11-14 days after enrollment, respectively.

A baseline questionnaire conducted at enrollment collected participant and household sociodemographic information, care-seeking preferences and attitudes toward phone-specific elements of the study protocol. During the same visit, field workers recorded participant household location using a Garmin e-Trex GPS device. Participants were then visited monthly over six months and administered a questionnaire about recent childhood illness and any subsequent care-seeking. After completing this questionnaire, participants with one or more health facility visits detected through the GPS approach were administered a supplementary questionnaire on these visits (see below). Field workers assessed participant compliance at each follow-up visit and provided support whenever required.

### Participant mobility tracking and detection of health facility visits

Participant smartphones were pre-installed with TrackCare, an application developed for this study to record the phone’s location at one-minute intervals and transmit these data hourly to a central study server. Details of the application’s development and implementation are described elsewhere, including GPS data quality (mean observation time of 152 days [84% completeness], median accuracy of 12 m), and participant compliance with phone-specific protocols (79% overall) [33].

Participant GPS data were analyzed to detect potential health facility visits using a parameter-based geofencing approach. The approach defined a radius around each identified facility location, *d_max_*, within which participant coordinates would be considered as within range of the specified facility. Sequential coordinates within range of the facility for longer than a minimum duration threshold, *t_min_*, were classified as a facility visit. Recognizing the potentially noisy nature of GPS data, we allowed coordinates to temporarily appear outside the range of a health facility for an interval, *t_int_*, provided that the coordinates subsequently reentered the facility range. Clusters of GPS data meeting these criteria were classified as potential health facility visits with the time associated with the earliest coordinate designated as the visit start time and the time associated with the last coordinate designated as the visit end time.

Optimal parameter values were determined through field-worker simulated health facility visits. A random sample of 15 facilities was selected from those identified during the facility census. Two field workers were each given one smartphone, preinstalled with TrackCare, and instructed to visit every location as if they were taking a child for care. These simulated visits lasted 10 minutes each. This time period was decided to be representative of the actual visit duration for most episodes of non-severe illness. All GPS data generated on the day of the simulation exercise were analyzed to detect health facility visits according to 432 parameter combinations: *d_max _=* each 5 m from 15-50 m, *t_min _=* each minute from 0-5 minutes, and *t_int _=* each minute from 2-10 minutes. Combination-specific results were compared to field workers’ known health facility visit status through two-by-two contingency tables with columns indicating true visit status and the rows indicating the combination-specific visit status. The optimal parameter combination was selected to maximize the area under the receiver operator characteristic curve (AUC), a combined measurement of sensitivity and specificity. Ties among similarly performing parameter combinations were decided in favor of the more conservative parameter values.

We applied the identified parameter combination to the prospective detection of health facility visits participant within participant GPS data during study implementation. The day of each scheduled follow-up visit we analyzed the previous two weeks of participant GPS data to detect health facility visits. When one or more visits were detected, a list of detected visits was prepared and distributed to field workers. This list included the facility location, visit date, start time, and end time. If multiple visits were detected to the same location on a given day, one row was included on the list for each detected visit. Field workers reviewed these lists with participants at the end of each follow-up visit, asking participants to confirm whether each detected visit actually occurred and, if so, whether it was related to care-seeking for childhood illness. Participant responses were subsequently entered electronically into a list-specific Excel file (Microsoft Inc, Seattle, WA, USA).

### Measures and statistical analysis

The overall performance of the GPS-based visit detection approach was assessed based on the proportion of all detected visits that were subsequently confirmed by participants during the prompted recall survey, regardless of visit purpose. Logistic regression models were applied to further explore the factors associated with the detection of false positive visits with each detected visit representing a single observation. Analysis was restricted to visits detected during participant follow-up periods with at least 50% GPS data completeness (ie, participants submitted GPS coordinates for at least 10 080 of the 20 160 total minutes during each follow-up period). Sensitivity analysis considered alternative data completeness thresholds (no threshold, >75%, >90%).

**Figure 2:**
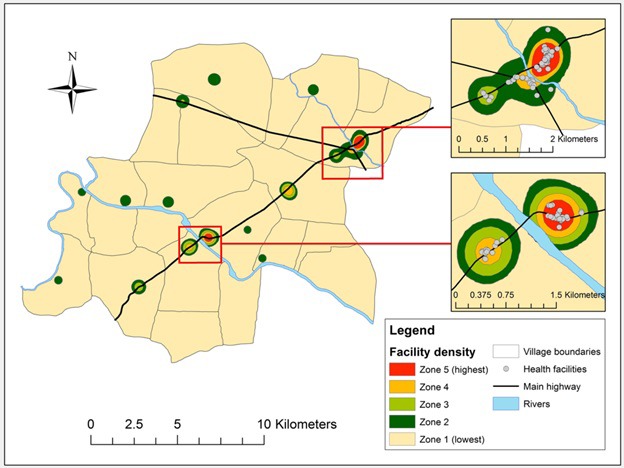
Map of Vadu Health and Demographic Surveillance System study area with health facility density zones and inset maps displaying areas of high health facility density. Note: Kernel density estimation based on 500-m bandwidth. Zone 1 includes 97% (224.7 km^2^) of the study area, Zone 2 includes 2% (4.6 km^2^), Zone 3 includes 1% (1.7 km^2^), and Zones 4 and 5 include less than 1% each (0.8 and 0.5 km^2^, respectively).

We explored the association between visit confirmation status and various aspects of health facility density. This association was modeled according to kernel density estimation (KDE), a geospatial analysis technique for exploring the distribution of features (eg, health facilities) in space [34]. The approach overlays a cone-shaped probability density function on each point, the density of which decreases with distance from the center. The width of the kernel function depends on the user-defined bandwidth, which establishes the radius around the point within which the density function is contained. Where two density functions overlap (eg, due to multiple features located near one another), the density value in the overlapping section is the sum of the individual functions. Selecting the appropriate bandwidth is key, with excessively low or high values leading to under- or over-smoothing of the data, both of which interfere with the identification of trends within the data. We evaluated seven bandwidths ranging from 100m to 2500m, classifying the resulting maps into quintiles (zones) of facility density [35]. Visual inspection identified 500m as the optimal bandwidth (**Figure 2**), as this value eliminated the noise present at lower values while preserving the important regions lost to over-smoothing at higher values (Figures S1-S7 in the **Online Supplementary Document**). Of the 232.2 km^2^ included in the study area, Zone 1 accounts for 97% (224.7 km^2^), Zone 2 accounts for 2% (4.6 km^2^), Zone 3 accounts for 1% (1.7 km^2^), and Zones 4 and 5 each account for less than 1% (0.8 and 0.5 km^2^, respectively).

Explanatory variables included characteristics of the detected visit, participant location characteristics, and sociodemographic characteristics of participants. Characteristics of the detected visit included duration, timing (overnight, other), proximity to participant residence and main highway, detected location sector (public, private) and type (hospital/clinic, other), location residence (urban, rural), and health facility density zone at detected location (Zone 1, other). Participant location characteristics included residence (urban, rural), proximity to main highway, health facility density zone of residence, and average health facility density zone for GPS coordinates submitted during follow-up period. Participant sociodemographic characteristics included maternal age, education and employment status; previous household smartphone ownership; and household socioeconomic status (SES), defined according to the principal components analysis approach used by the Demographic and Health Survey [36].

Proximity to the main highway and health facility density zones at both the location and participant level were calculated using ArcGIS version 10.3 (Environmental Systems Research Institute, Redlands, CA, USA) [37]. Average participant health facility density zone was calculated by plotting all participant GPS coordinates submitted during each follow-up period, identifying the corresponding health facility density zone for each coordinate, and computing the average value for the period. While the health facility density zone linked with each participant residence is likely to be highly correlated with the average zone during the follow-up period, the latter measure should provide a more accurate measurement of the participant’s exposure to areas of high and low health facility density.

We estimated the unadjusted and adjusted associations between individual predictors and visit confirmation status through bivariate and multivariable logistic regression models, respectively, with standard errors adjusted for clustering among observations from the same participant. All variables demonstrating a marginally significant association with visit confirmation status in bivariate analysis (*P* < 0.10) were considered for inclusion in the multivariable model. The final set of variables included in the model was guided by the Akaike’s information criterion and the Hosmer-Lemeshow goodness-of-fit test.

### Ethical considerations

All participants provided written consent prior to group assignment. Before obtaining consent, field workers informed participants that those assigned to the phone group would receive a smartphone and would be allowed to keep the device regardless of study completion. Various safeguards ensured the privacy of participant location data. Data stored on participant phones were saved in an encrypted database and were erased once they had been successfully transferred to a central study server. The study protocol was approved by the ethics committees of the University of Edinburgh and K.E.M. Hospital Research Centre, Pune (Study ID No. 1415).

## RESULTS

We enrolled 200 mothers from June to September 2015. The baseline characteristics for mothers are presented in **Table 1**, stratified by health facility density zone of participant residence. One participant withdrew from the study before completing the first follow-up visit and was excluded from analysis. Average participant age was 25.3 years (standard deviation (SD) = 3.3) with 10.9 completed years of schooling (SD = 2.7). Current employment was reported by 28% of participants and was highest among participants located in density zone 1. Previous smartphone ownership was reported by 69% of participants. Two thirds of households resided in one of the four urban villages located along the main highway, while the remaining third were distributed among the 18 rural villages. The proportion of households located in urban villages was lowest among participants in density zone 1 and increased by zone. Median distance from a participant’s residence to the main highway was 0.5 km (interquartile range (IQR) = 0.2-2.5) with a median of one health facility located within 500 m (IQR = 0-11). Participants in higher density zones were located nearer to the highway and had a greater number of health facilities nearby.

**Table 1:** Baseline participant characteristics by health facility density zone*

Characteristic	Total (N = 199), N (%)	Zone 1, N = 123), N (%)	Zone 2, (N = 29), N (%)	Zone 3, (N = 19), N (%)	Zone 4, (N = 20), N (%)	Zone = 5, (N = 8), N (%)	*P-*value
Maternal age, mean (SD)	25.3 (3.3)	25.3 (3.3)	25.4 (3.4)	24.4 (3.9)	25.7 (2.7)	25.5 (2.3)	0.80
Maternal education (years), mean (SD)	10.9 (2.7)	10.9 (2.6)	10.5 (2.3)	11.2 (3.5)	10.6 (3.0)	11.9 (2.5)	0.71
Maternal employment	54 (28%)	43 (36%)	4 (14%)	3 (16%)	2 (10%)	2 (25%)	0.04
**Wealth quintile:**							
1 (Lowest)	40 (20%)	18 (15%)	9 (31%)	6 (32%)	4 (20%)	3 (38%)	0.24
2	40 (20%)	23 (19%)	8 (28%)	4 (21%)	5 (25%)	0 (0%)	
3	40 (20%)	22 (18%)	7 (24.1%)	5 (26.3%)	4 (20%)	2 (25%)	
4	40 (20%)	30 (24%)	4 (13.8%)	1 (5.3%)	4 (20%)	1 (13%)	
5 (Highest)	39 (20%)	30 (24%)	1 (3.4%)	3 (15.8%)	3 (15%)	2 (25%)	
Previous smartphone ownership	132 (69%)	85 (71%)	13 (50%)	15 (79%)	13 (65%)	6 (75%)	0.21
**Participant residence:**							
Rural	67 (34%)	61 (50%)	6 (21%)	0 (0%)	0 (0%)	0 (0%)	<0.001
Urban	132 (66%)	62 (50%)	23 (79%)	19 (100%)	20 (100%)	8 (100%)	
Distance to highway (km), median (IQR)	0.5 (0.2, 2.5)	1.6 (0.5, 3.1)	0.3 (0.2, 0.6)	0.2 (0.1, 0.3)	0.1 (0.0, 0.2)	0.1 (0.1, 0.2)	<0.001
Facility locations within 500m, median (IQR)	1 (0, 11)	0 (0, 1)	7 (6, 13)	13 (7, 18)	18 (14, 24)	32 (26, 40)	<0.001

SD – standard deviation, km – kilometer, IQR – interquartile range, m – meter

*Health facility density zones determined according to kernel density estimation using 500-m bandwidth.

A total of 196 provider locations were identified, including seven public sector health facilities, 93 private hospitals and clinics, and 68 pharmacies. Facility premises were shared by 29 providers, resulting in 167 unique provider locations (eg, private hospital on first floor with a pharmacy on ground floor). Baseline location characteristics stratified by density zone are presented in **Table 2**. Locations were most commonly in the private sector (81%) and either hospitals or clinics (59%), though this pattern was reversed among density zone 1 (94% public; 76% non-hospital/clinic) and was less pronounced in density zone 2 (61% private; 52% hospital/clinic). Most facilities were located near the main highway (median distance 0.1 km; IQR = 0.0-0.3) with facilities in zones 3-5 located nearer the highway than facilities in zones 1-2. A high degree of clustering of health facilities was observed overall with a median of 17 other facilities located within 500 m of each facility (IQR = 4-25). This was lowest in zone 1 (median 0, IQR = 0-0) and increased proportionally with density zone until zone 5 (median 34, IQR = 25-35).

**Table 2:** Baseline facility characteristics by health facility density zone

Characteristic	Total (N = 167), N (%)	Zone 1 (N = 17), N (%)	Zone 2 (N = 23), N (%)	Zone 3, (N = 36), N (%)	Zone 4 (N = 34), N (%)	Zone = 5 (N = 67), N (%)	*P*-value
**Sector:**							
Public	31 (19%)	16 (94%)	9 (39%)	3 (12%)	0 (0%)	3 (4%)	<0.001
Private	136 (81%)	1 (6%)	14 (61%)	23 (88%)	34 (100%)	64 (96%)	
**Type, general:**							
Hospital/clinic	99 (59%)	4 (24%)	12 (52%)	17 (65%)	23 (68%)	43 (64%)	0.021
Other*	68 (41%)	13 (76%)	11 (48%)	9 (35%)	11 (32%)	24 (36%)	
**Type, specific:†**							
Rural hospital	1 (<1%)	0 (0%)	1 (4%)	0 (0%)	0 (0%)	0 (0%)	<0.001
Primary health centre	1 (<1%)	1 (6%)	0 (0%)	0 (0%)	0 (0%)	0 (0%)	
Sub-centre/ANM	5 (3%)	3 (18%)	1 (4%)	1 (4%)	0 (0%)	0 (0%)	
Anganwadi/ICDS centre	24 (14%)	12 (71%)	7 (30%)	2 (8%)	0 (0%)	3 (4%)	
NGO/trust hospital/clinic	1 (<1%)	0 (0%)	1 (4%)	0 (0%)	0 (0%)	0 (0%)	
Pvt. hospital	49 (29%)	0 (0%)	2 (9%)	10 (38%)	12 (35%)	25 (37%)	
Pvt. doctor/clinic	42 (25%)	0 (0%)	7 (30%)	6 (23%)	11 (32%)	18 (27%)	
Pharmacy/drugstore	40 (24%)	0 (0%)	1 (4%)	7 (27%)	11 (32%)	21 (31%)	
Shop	4 (2%)	1 (6%)	3 (13%)	0 (0%)	0 (0%)	0 (0%)	
**Location:**							**<0.001**
Rural	34 (20%)	10 (59%)	19 (83%)	5 (19%)	0 (0%)	0 (0%)	
Urban	133 (80%)	7 (41%)	4 (17%)	21 (81%)	34 (100%)	67 (100%)	
Distance to highway (km), median (IQR)	0.1 (0.0, 0.3)	1.5 (0.9, 4.1)	2.7 (1.8, 3.8)	0.0 (0.0, 0.1)	0.0 (0.0, 0.1)	0.0 (0.0, 0.1)	<0.001
Other locations within 500m, median (IQR)	17 (4, 25)	0 (0, 0)	2 (1, 3)	6 (5, 11)	17 (13, 18)	34 (25, 35)	<0.001

NGO – non-govermental organisation, ANM – auxiliary nurse midwife, ICDS – Integrated Child Development Services, IQR – interquartile range

*Includes Anganwadi/ICDS centre, Pharmacy/drugstore, and Shop.

†In the case of 23 private hospitals and 5 private doctors/clinics, the same location coordinate was shared with a pharmacy/drugstore. The table presents the primary location type. For these shared location spaces, the primary type would be the private hospital or clinic.

Two field workers simulated visits to a total of 15 health facilities while carrying smartphones installed with the TrackCare application. Location data generated during the simulation exercise were analyzed according to 432 combinations of distance, time, and interval parameters (Table S1 in the **Online Supplementary Document**). The optimal combination specified *d_max _=* 25 m, *t_min_* = 3 minutes, and *t_int_* = 8 minutes. This combination correctly identified visits to 14 of the 15 simulated visits (**Table 3**). However, this combination incorrectly identified visits at four facilities without a simulated visit from among the 117 eligible facilities not visited during the simulation. Sensitivity, specificity, AUC, negative predictive value, and accuracy all exceed 90%. Positive predictive value, the proportion of visits identified by the detection algorithm that actually occurred, is comparatively lower at 78%.

**Table 3:** GPS visit detection performance when applied to simulated facility visit data, optimal parameter combination (*d_max _=* 25 m, *t_min _=* 3 min, and *t_int _=* 8 min)*

		Field worker visit status		
**Facility visited**	**Facility not visited**	**Total**
**GPS visit detection status**	Visit detected	14	4	18
	Visit not detected	1	98	99
	Total	15	102	117

GPS – global positioning system

*Sensitivity = 93%, Specificity = 96%, Area under receiver operating characteristic curve = 95%, Positive predictive value = 78%, Negative predictive value = 99%, Accuracy = 96%.

A summary of participant follow-up visits is presented in **Table 4**. Field workers completed 1098 follow-up visits (8% loss to follow-up) with completion higher during earlier follow-up visits. Participant GPS data corresponding to each follow-up period were analyzed to detect health facility visits, identifying one or more visits during 793 follow-up visits (72%). Prompted recall surveys were completed during 694 of these visits (88%), constituting the evaluable sample for the remaining analysis. The proportion of participant follow-up visits where a prompted recall survey was indicated but was not completed increased during follow-up visits 5 and 6 due to server-related issues. While participant data were continually transferred to the central study server, a delay in relaying these data to the local study site resulted in field workers receiving the lists of GPS-detected visits after completion of the corresponding follow-up visits.

**Table 4:** Participant follow-up visits completed, with GPS visits indicated, and with prompted recall completed

	Follow-up visits planned, N	(B) Follow-up visits completed, N (% of A)	(C) Follow-up visits with GPS visit indicated N (% of B)	(D) Follow-up visits with prompted recall completed, N (% of C)	(E) Summary data from follow-up visits with prompted recall completed	
**Completeness of GPS data, Median (IQR)**	**GPS-detected visits per list, Median (IQR)**
All visits	1,194	1098 (92%)	793 (72%)	694 (88%)	78% (60%-92%)	8 (3-27)
Visit 1	199	191 (96%)	150 (79%)	144 (96%)	80% (61%-92%)	8 (4-17)
Visit 2	199	191 (96%)	139 (73%)	138 (99%)	80% (56%-92%)	7 (3-27)
Visit 3	199	183 (92%)	136 (74%)	132 (97%)	78% (60%-91%)	8 (3-30)
Visit 4	199	180 (90%)	129 (72%)	113 (88%)	70% (55%-88%)	9 (4-22)
Visit 5	199	174 (87%)	114 (66%)	73 (64%)	78% (62%-94%)	12 (4-31)
Visit 6	199	179 (90%)	125 (70%)	94 (75%)	74% (59%-92%)	7 (3-26)

GPS – global positioning system, IQR – interquartile range

The visit detection algorithm identified a total of 22 251 possible health facilities visits across all study visits with participants confirming 440 (2%) of these visits as having occurred. The remaining visits represent false positives. Characteristics of each visit stratified by confirmation status are presented in **Table 5**. Median visit duration was 13 minutes (IQR = 7-28) with no significant difference by confirmation status. Visits occurring overnight accounted for 28% of all detected visits but only 12% of confirmed visits (*P* < 0.001). The median distance between the facility at which each visit was detected and the corresponding participant’s household was 0.1 km (IQR = 0.0-0.6). Confirmed visits tended to be further from the participants household than visits that were not confirmed (1.4 vs 0.1km, *P* < 0.001). Hospitals and clinics accounted for 59% of all detected visits with a larger proportion of confirmed visits occurring at hospitals and clinics than non-confirmed visits (76% vs 59%, *P* < 0.001). The performance of the visit detection algorithm varied by provider type, with 79% of visits detected at the rural hospital confirmed (11 visits) and both visits detected at the primary health center were confirmed. In contrast, none of the 854 combined visits detected at any of the government sub-centers, the NGO hospital, or included shops were confirmed. Private sector facilities accounted for a greater proportion of confirmed visits than non-confirmed visits (95% vs 91%, *P* = 0.003). Detected visits were primarily at facilities located in urban villages with no significant difference by confirmation status.

**Table 5:** Characteristics of detected visits by confirmation status

Characteristic	All detected visits (N = 22 251), N (%)	True positives (N = 440), N (%)	False positives (N = 21 811), N (%)	*P*-value
Duration (minutes), median (IQR)	13 (7, 28)	13 (7, 31)	13.1 (7, 28)	0.49
Visit occurred overnight*	6159 (28%)	54 (12%)	6105 (28%)	<0.001
Distance from participant residence (km), median (IQR)	0.1 (0.0, 0.6)	1.4 (0.4, 3.5)	0.1 (0.0, 0.5)	<0.001
Distance from highway (km), median (IQR)	0.0 (0.0, 0.1)	0.0 (0.0, 0.1)	0.1 (0.0, 0.2)	<0.001
**Facility sector:**				
Public	2071 (9%)	23 (5%)	2048 (9%)	0.003
Private	20 180 (91%)	417 (95%)	19 763 (91%)	
Facility type, general:				
Hospital/clinic	13 206 (59%)	336 (76%)	12 870 (59%)	<0.001
Other^†^	9045 (41%)	104 (24%)	8941 (41%)	
**Facility type, specific:**				
Rural hospital	14 (<1%)	11 (3%)	3 (<1%)	<0.001
PHC	2 (<1%)	2 (<1%)	0 (0%)	
Sub-centre/ANM	229 (1%)	0 (0%)	229 (1%)	
Anganwadi/ICDS centre	1826 (8%)	10 (2%)	1816 (8%)	
NGO or trust hospital/clinic	302 (1%)	0 (0%)	302 (1%)	
Private hospital	6302 (28%)	277 (63%)	6025 (28%)	
Private doctor/clinic	6357 (29%)	46 (10%)	6311 (29%)	
Pharmacy/drugstore	6896 (31%)	94 (21%)	6802 (31%)	
Shop	323 (1%)	0 (0%)	323 (1%)	
**Facility location:**				
Rural	2055 (9%)	41 (9%)	2014 (9%)	0.95
Urban	20 196 (91%)	399 (91%)	19 797 (91%)	
**Health facility density zone:**				
1 (Lowest density)	1605 (7%)	11 (3%)	1594 (7%)	<0.001
2	1229 (6%)	44 (10%)	1185 (5%)	
3	1968 (9%)	47 (11%)	1921 (9%)	
4	5648 (25%)	51 (12%)	5597 (26%)	
5 (Highest density)	11 801 (53%)	287 (65%)	11 514 (53%)	

IQR – interquartile range, NGO – non-governmental organisation, PHC – primary health centre, ANM – auxiliary nurse midwife, ICDS – Integrated Child Development Services

*Visit began after 21:00 and ended before 07:00.

†Includes Anganwadi/ICDS centre, Pharmacy/drugstore, and Shop.

The results of the bivariate and multivariable logistic regression models are presented in **Table 6**. The final model included the distance between the facility at which the visit was detected and the participant’s residence, the facility type (hospital/clinic, other), the density zone of the facility (zone 1, other), average participant density zone during follow-up, maternal employment status and follow-up visit number. False positives were more likely among visits detected nearby participant residences with the likelihood of detecting a false positive decreasing inversely with distance from a participant (odds ratio (OR) = 0.89, 95% confidence interval (CI) = 0.82-0.97). Visits detected at facilities other than hospitals and clinics were more likely to be false positives (OR = 5.29, 95% CI = 1.65-4.67), as were visits detected at facilities located in the zone of lowest health facility density (OR = 5.29, 95% CI = 1.74-16.05). The likelihood of detecting false positives also increased as participants spent more time in areas of increased health facility density (OR = 2.29, 95% CI = 1.62-3.25). Maternal employment status was associated with detected visits being false positives (OR = 3.78; 95% CI = 1.79-7.97) as was follow-up visit number (OR = 1.19; 95% CI = 1.02-1.39). The time of day when the visit was detected and the density zone within which participant households were located were significantly associated with confirmation status in bivariate analysis but were excluded from the final model due to collinearity with other predictors. In the case of household density zone, we compared models with household density zone and the average zone of participant GPS points during follow-up and found that the latter resulted in improved model fit.

**Table 6:** Unadjusted and adjusted associations with false positive health facility visit detection

Characteristic	Unadjusted OR (95% CI)	Adjusted OR (95% CI)
**Characteristics of the detected visit:**		
Duration, minutes	1.00 (1.00-1.00)	-
Visit occurred overnight*	3.32 (1.55-7.13)‡	-
Distance from participant residence, km	0.78 (0.71-0.85)‡	0.89 (0.82-0.97)§
Distance from highway, km	1.09 (0.86-1.39)	-
Location is private sector	0.58 (0.23-1.42)	-
**Location type:**		
Hospital/clinic	REF	REF
Other†	2.57 (1.44-4.57)‡	2.78 (1.65-4.67)‡
Location in density zone 1	3.23 (1.06-9.85)§	5.29 (1.74-16.05)‡
Location in urban village	1.01 (0.38-2.7)	-
**Participant location characteristics:**		
Urban village	2.74 (1.16-6.48) §	-
Distance from highway, km	0.8 (0.64-0.99) §	-
**Zone of participant residence:**		
1 (lowest density)	REF	-
2	1.88 (0.82-4.33)	-
3	8.83 (1.55-50.33)§	-
4	4.32 (1.29-14.47)§	-
5 (highest density)	11.04 (2.08-58.73)‡	-
Average zone during follow-up	2.04 (1.51-2.75)‡	2.29 (1.62-3.25)‡
**Participant sociodemographic characteristics:**		
Maternal age, years	1.03 (0.91-1.18)	-
Maternal education, completed years	1.09 (0.92-1.3)	-
Maternal employment	2.61 (0.89-7.69)‖	3.78 (1.79-7.97)‡
Previous smartphone ownership	1 (0.36-2.81)	-
**Household wealth quintile:**		
1 (lowest)	REF	-
2	1.42 (0.4-5.04)	-
3	0.72 (0.19-2.72)	-
4	0.66 (0.18-2.47)	-
5 (highest)	1.45 (0.32-6.64)	-
**Follow-up visit number**	1.16 (0.97-1.38)	1.19 (1.02-1.39) §

OR – odds ratio, CI – confidence interval

*Visit began after 21:00 and ended before 07:00.

†Includes Anganwadi/ICDS centre, Pharmacy/drugstore, and Shop.

‡*P* < 0.01.

§*P* < 0.05.

‖*P* < 0.10; Note: confidence intervals adjusted for clustering among repeated observations from the same participant.

Sensitivity analyses compared different cutoffs of GPS completeness and various specifications of the KDE bandwidth. Point estimates were consistent across all levels of GPS completeness, though confidence intervals for some associations that were significant at more permissive levels of data inclusion became non-significant as more data were excluded (Figure S8 in the **Online Supplementary Document**). Point estimates and confidence intervals for facility type, maternal employment, and visit number were consistent between varying specifications of the KDE bandwidth (Figure S9 in the **Online Supplementary Document**). Point estimates and confidence intervals for KDE-derived variables were highly sensitive to the bandwidth specification. The modeled effect size of each variable was attenuated as the bandwidth is increased.

## DISCUSSION

This study developed and implemented a parameter-based geofencing approach to detect health facility visits from passively collected participant GPS data. While similar approaches set parameters based on *a priori* assumptions of their optimal value, this study benefited from a calibration exercise where parameter values were determined using locally simulated health facility visits. The results of this calibration were then applied to the prospective detection of health facility visits among a cohort of 199 mothers with young children over six months of follow-up. The overall performance of the GPS-based approach, measured as the proportion of all detected visits that were subsequently confirmed by participants during monthly prompted recall visits (440 confirmed of 22 251 detected), was low at 2%.

Researchers using algorithms to detect visited locations passively have struggled to balance detection of all true visits while minimizing the detection of false visits, though our results indicate lower performance than what has been reported elsewhere. Paz-Soldan et al. analyzed data collected from GPS trackers to detect visits to a variety of location types in a Peruvian city, with 47% of visits to health facilities and 41% of visits overall subsequently in participant interviews [20]. Nguyen et al. developed a GPS-based approach to detect hospitalizations across the United States, with participants confirming 65% of all detected hospitalizations [19]. In both cases, a more conservative approach was applied to the classification of visits than was performed in our study.

We applied three parameters to the detection of health facility visits: distance from the health facility (*d_max _=* 25m), duration within that distance (*t_min _=* 3mins), and interval during which points could temporarily appear outside that distance and still be considered part of the visit (*t_int _=* 8mins). In contrast, the combination applied by Paz-Soldan et al. set *d_max _=* 20m, *t_min_* = 30mins, and *t_int_* = 15mins [20]. The distance and duration threshold are more conservative than the values applied in our study, requiring that an individual be both nearer to a location and remain there over a longer period of time before being considered to have visited that location. Nguyen et al. specify an even higher duration threshold of 4 hours, though information is not provided on other parameters [19]. Three quarters of visits detected in our study had a duration less than 30 minutes with no difference between visits by confirmation status, so it is unclear whether applying a higher duration parameter would result in improved performance.

While our selected parameter combination performed well when applied to field worker-simulated health facility visits (PPV = 78%), this combination resulted in a fifty-fold overestimation of health facility visits when applied to participant GPS data. This suggests a systematic difference between the simulation data set and participant data collected throughout the study duration. Field workers were directed to visit several facilities over one day of testing, typically traveling directly from one facility to the next without spending time on other activities in the area surrounding the health facility. Field worker mobility during the simulation is therefore less likely to be representative of participants when moving through the study area. Consequently, field workers were less likely to engage in activities that might be associated with the detection of false positives (eg, shopping at a location nearby a facility). The absence of such activities from the calibration data set may have skewed the resulting parameter values. Also, the relatively short duration of each simulated visit (10 minutes) may have biased our duration value toward the value of three minutes, likely contributing to the high number of false positives detected. The combination of these factors may explain in part why the parameter combination performed well within the simulation exercise but poorly when applied to participant data. Future calibration efforts should include a broader range of simulated activities and make additional efforts to ensure that simulated data are representative of the participant behaviors under study. One recommendation for such an activity would be to enroll a small sample of participants, provide them with GPS-enabled smartphones, and ask that they also document their travel behavior (eg, through a travel diary). Such information could be used to train the detection algorithm to more accurately classify participant GPS data within the specific context of the study area.

Our analysis of confirmed visits provides valuable insight into the importance of study context. We identified five zones of health facility density with the zone of highest density including 40% of all identified health facilities. This zone included two regions with a combined area of 0.5 km^2^. Participants living in this zone represented only 4% of the study population yet accounted for 30% of all detected visits. Participant location was significantly associated with visit confirmation status for all measures examined in the unadjusted analysis. Visits were more likely to be false positives among participants residing in urban villages, nearer to the highway, and in areas of greater health facility density, measured either according to household location or the zone where participants spent time. The last of these covariates was highly associated with visit confirmation status in the adjusted model. In this case, the likelihood that a detected visit was a false positive more than doubled with each one unit increase in the average zone where a participant spent her time. As a participant’s exposure to highly urbanized areas with a high health facility density increases so too does the probability that she will pass within range of a health facility long enough for her data to indicate a visit to the facility. This complicates the implementation of a GPS-based approach for detecting visits to health facilities and other locations of interest in urban areas.

We also noted a significant association between health facility type and confirmation status. Visits detected at facilities other than hospitals and clinics were nearly three times as likely to be false positives than were visits at hospitals and clinics. Private sector facilities account for nearly all hospitals and clinics, while private pharmacies account for most other facility types. Within our study context, private hospitals and clinics are often located in similar areas. The nearly 3-fold reduction in performance among non-hospitals and clinics is therefore likely due to characteristics beyond facility location. Separately, we noted high performance at the government-run rural hospital (79%) and the primary health centre (100%), though only 16 visits were detected between the two. Both facilities are offset from the main road and are located in areas of lower density. While these limited numbers should be interpreted with caution, they suggest certain contexts where a similar approach may perform well.

The study had several limitations. First, our study used a prompted recall survey to evaluate the performance of our visit detection algorithm. While this is a common approach for assessing the validity of GPS-based inferences, it involves a component of participant recall and may be subject to the same biases as survey-based methods [38]. Stopher et al. evaluated prompted recall within the context of a transportation study to identify travel mode and trip purpose, finding that prompted recall misclassified trip mode and trip purpose in about 10% and 20% of records, respectively [38]. The authors propose that wearable cameras may provide an alternative to prompted recall, though such an approach would not be feasible in our study for several reasons. Second, while our prompted recall approach allowed for the classification of detected health facility visits as true positives and false positives, it did not include any evaluation of instances where the GPS method identified no visit. This would have required the inclusion of an additional section where participants were asked to list any visits that occurred but were not among the GPS-detected visits. The inclusion of such a section was considered during study design but omitted due to concerns that this would further prime participants to monitor their care-seeking behavior and unnecessarily bias the results of the parent study validating maternal care-seeking recall. Without confirmation of true negatives, the calculation of standard diagnostic metrics was not possible (eg, sensitivity and specificity). Third, visit confirmation status was only collected for those visits detected according to the specified parameter combination, complicating the consideration of alternative parameter definitions after the fact. More conservative parameter values may result in fewer visits detected but evaluating its performance would only be possible where detected visits were a subset of those for which prompted recall data are available. Fourth, study implementation revealed instances where an improbably large number of visits were detected during specific participant follow-up periods (eg, >25 visits detected in previous two weeks). These lists were reviewed with participants according to the same protocol as all other lists, though their length may have resulted in non-standard completion of the prompted recall survey and potential outcome misclassification due either to participant fatigue or field worker improvisations. Given our aim of exploring the performance of our visit detection algorithm, we have retained all visits in our analysis. The association of many factors with these large lists were also of interest in our analysis (eg, proximity between participant residence and health facility) and their exclusion would have biased our results. Finally, we assumed that GPS data collected from participant smartphones were a valid measurement of participant movement throughout the study period. This assumption held as long as the phone traveled with the participant but was violated when the participant traveled without the phone or when the phone was given to someone other than the participant. This is a common challenge faced by studies using GPS and other remote tracking technologies. However, a recent study found the magnitude of misclassification resulting from participant non-compliance to be relatively low [39]. If a non-participant visited a health facility when carrying the phone, it is unlikely that any resulting GPS-based visit would be confirmed by the participant during prompted recall. While such instances would be rare, these would lower the calculated performance of the approach.

This study demonstrates the development and implementation of a GPS-based approach to detect health facility visits. Such approaches rely on externally defined parameter values, the selection of which is of critical importance. Differences in study settings provide no guarantee that the parameter combination applied in one setting will perform similarly in another. Future studies should consider the process by which these parameters are set. There may also be interest in exploring how machine learning and other statistical techniques can be applied to refine a visit detection algorithm during study implementation. This study has also demonstrated the challenges associated with detecting health facility visits in areas of high density. Such an approach may perform much better in a more rural setting with fewer health facilities and where they are located further from urban centers. While we provided participants with smartphones, several studies have collected GPS data using participants’ own smartphones [40,41]. As the discriminative capacity of visit detection algorithms increases, combining such an approach with data generated from participant-owned devices and publicly available spatial data could greatly expand the scale of current research into health-related mobility at relatively low cost.

## CONCLUSION

While many studies have explored the capacity for GPS-based visit detection, few studies calibrate parameter values according to locally generated data sets. This study demonstrates one process by which locally simulated health facility visits informed the selection of potential parameter values, though uncertainty remains regarding the suitability of this process within this specific research context. While these values demonstrated high performance when applied to the calibration data set they resulted in a fifty-fold overestimation of visits when applied to participant-generated data, suggesting a systematic difference between the two data sets. While overall performance was low we observed several factors associated with performance. High clustering of health facilities within urban centers complicated the detection of health facility visits within this setting. Researchers interested in applying a GPS-based visit detection method within a context of similarly high health facility density should carefully consider the challenges posed by such a setting.

## Additional material

Online Supplementary Document
