# Supplementary material for: Validating a GPS-based approach to detect health facility visits against maternal response to prompted recall survey
Source: J Glob Health. 2020 May 2;10(1):010602. doi: 10.7189/jogh.10.010602 (PMC7211413; doi:10.7189/jogh.10.010602)
Supplement: Online Supplementary Document [file jogh-10-010602-s001.pdf]

Appendix Tables and Figures

Figure A1: Map of Vadu HDSS with health facility density zones, 100m bandwidth

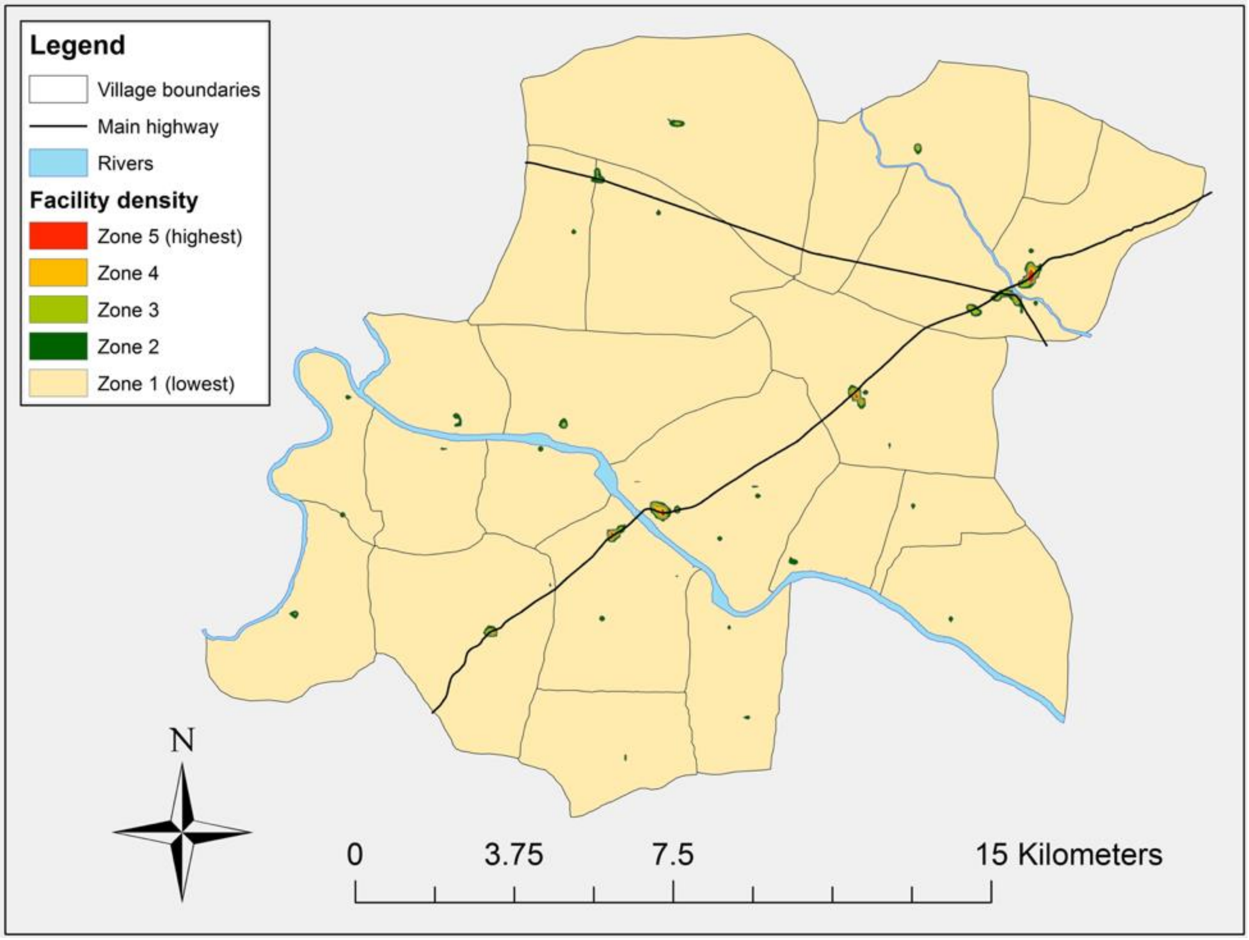

Figure A2: Map of Vadu HDSS with health facility density zones, 250m bandwidth

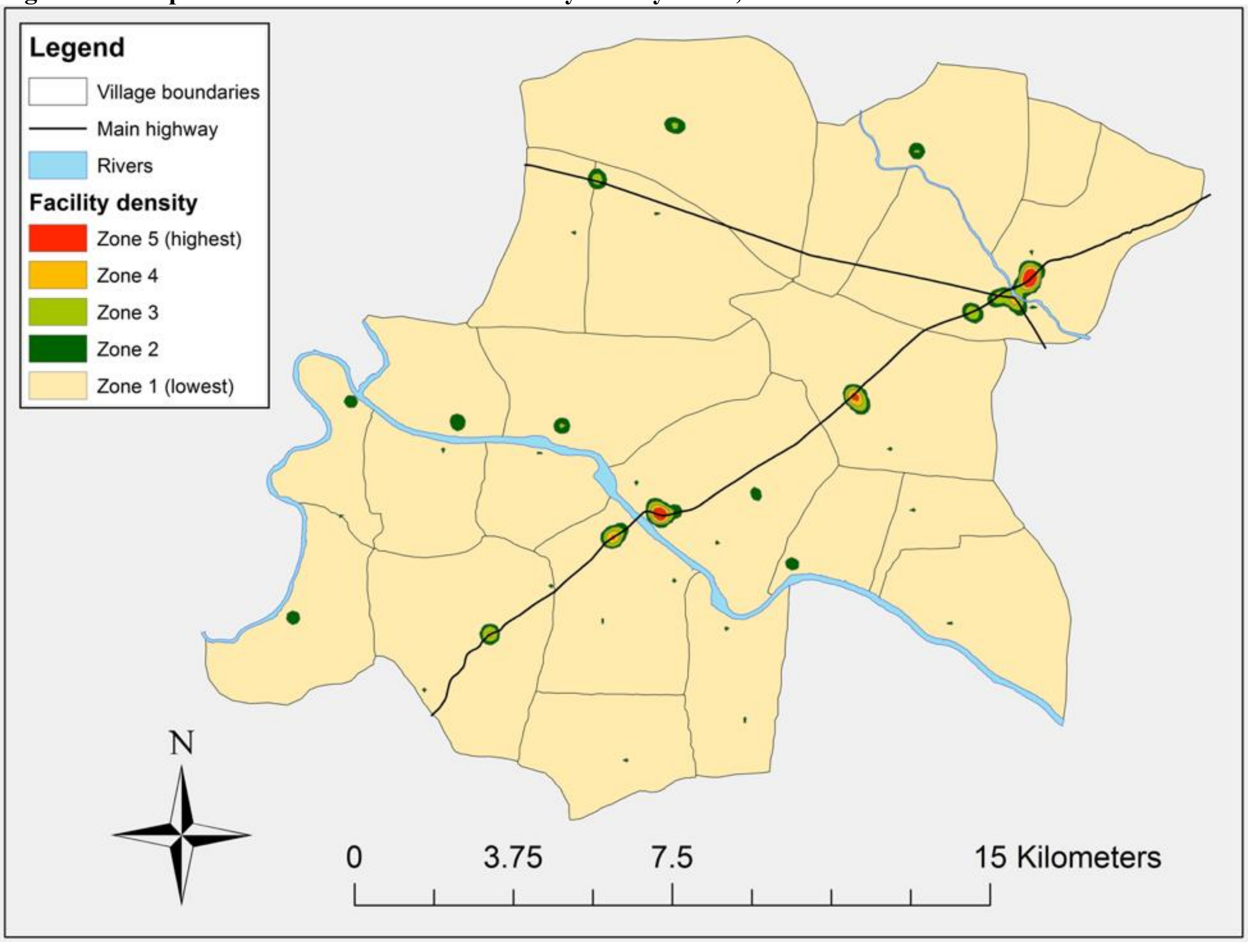

Figure A3: Map of Vadu HDSS with health facility density zones, 500m bandwidth

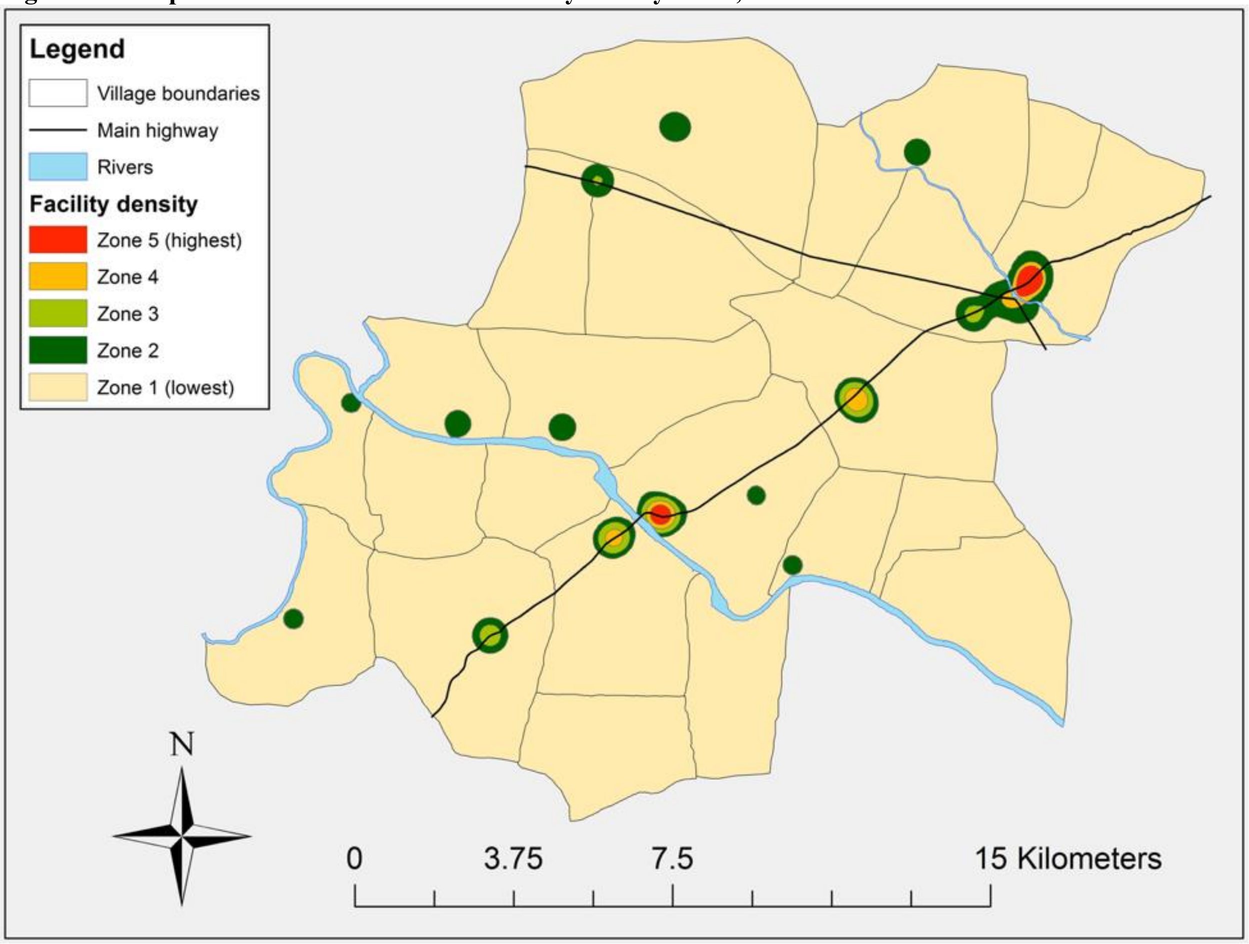

Figure A4: Map of Vadu HDSS with health facility density zones, 1000m bandwidth

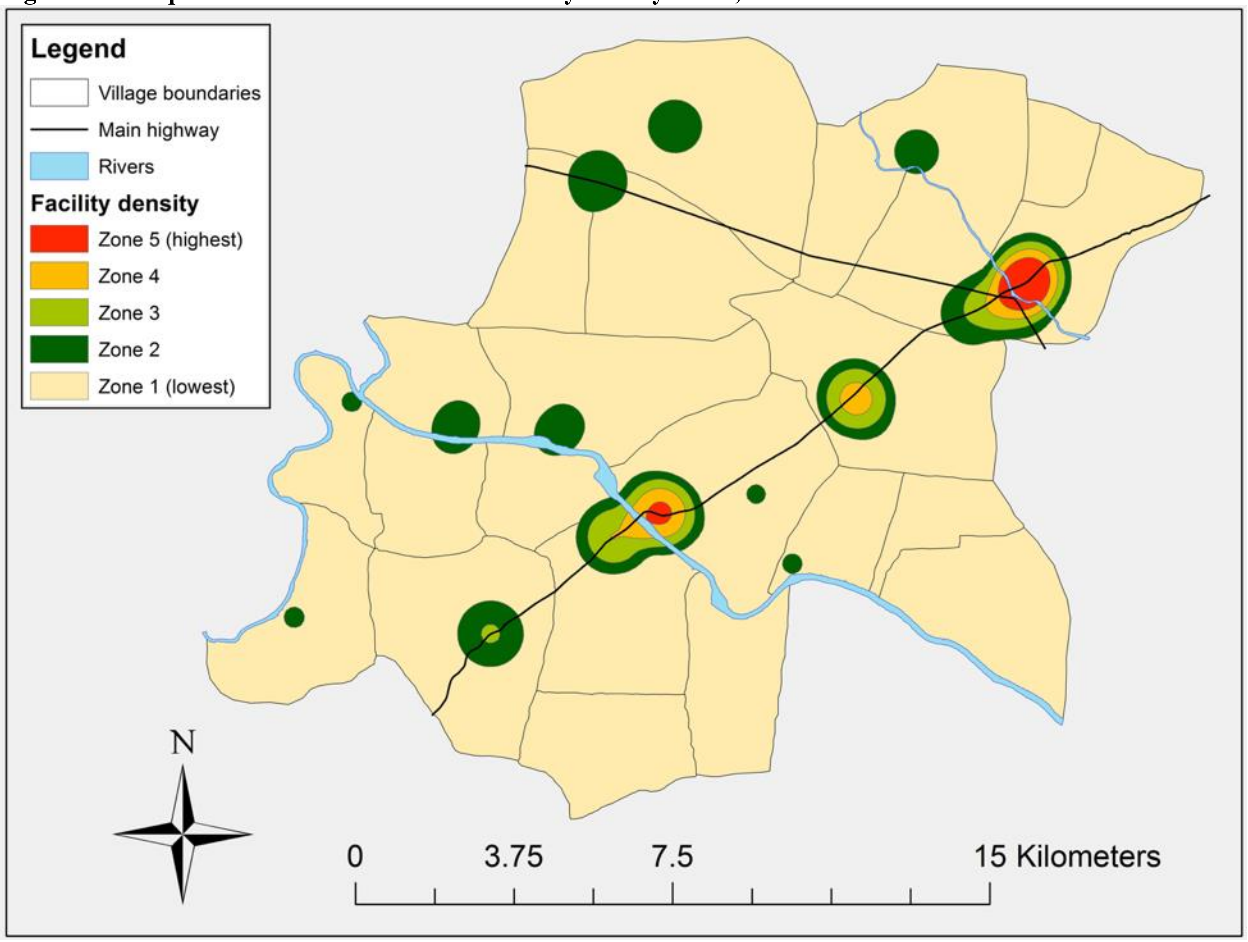

Figure A5: Map of Vadu HDSS with health facility density zones, 1500m bandwidth

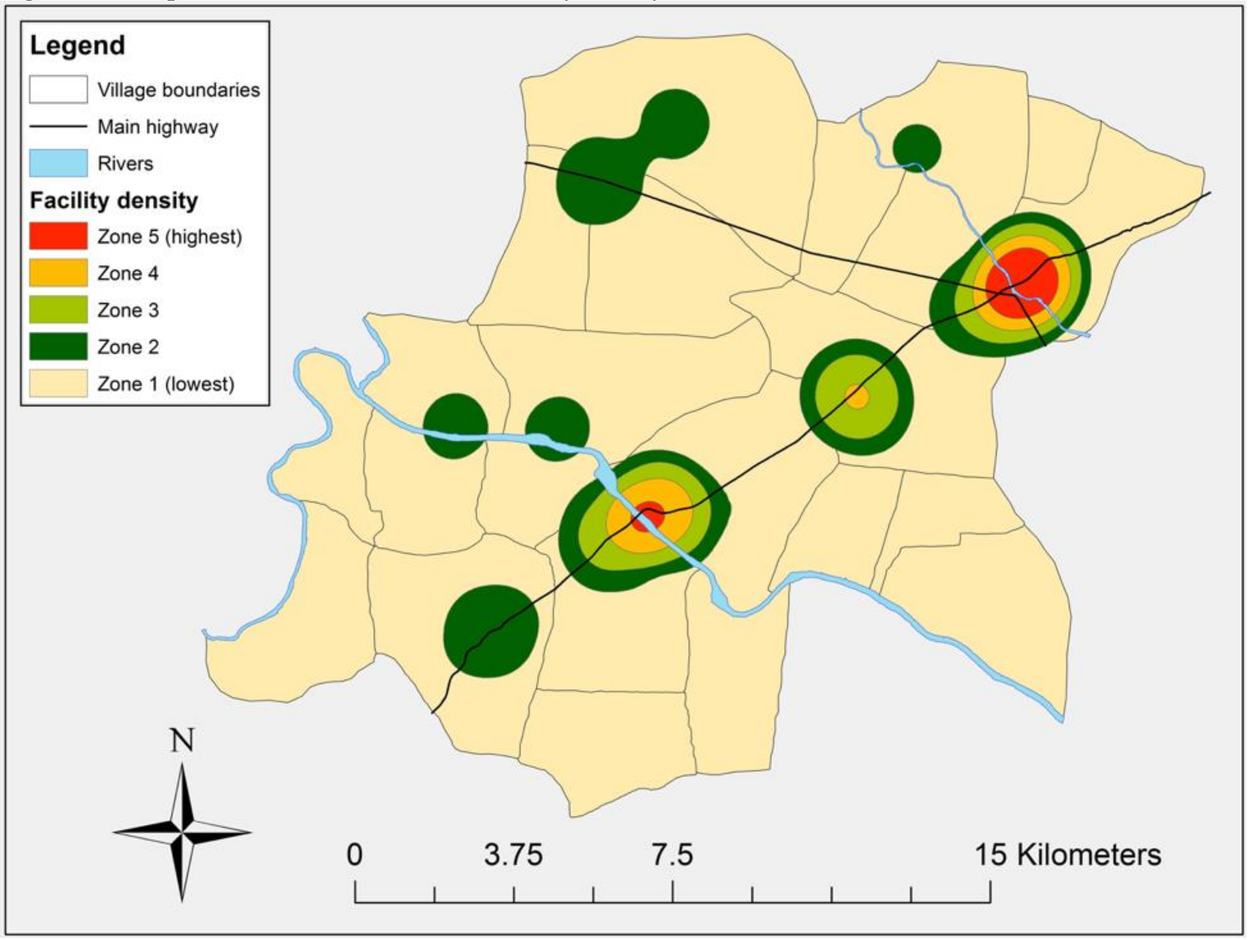

Figure A6: Map of Vadu HDSS with health facility density zones, 2000m bandwidth

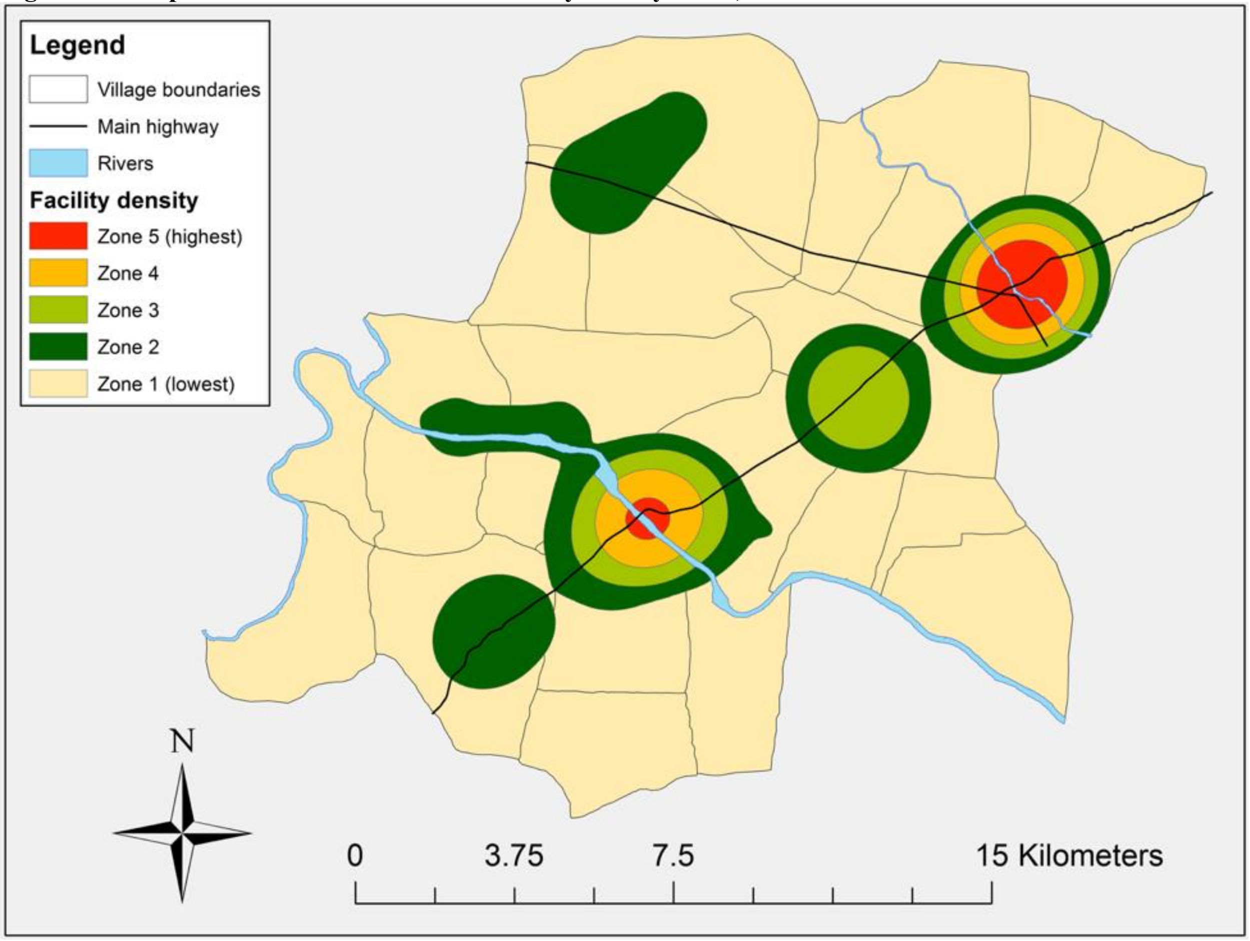

Figure A7: Map of Vadu HDSS with health facility density zones, 2500m bandwidth

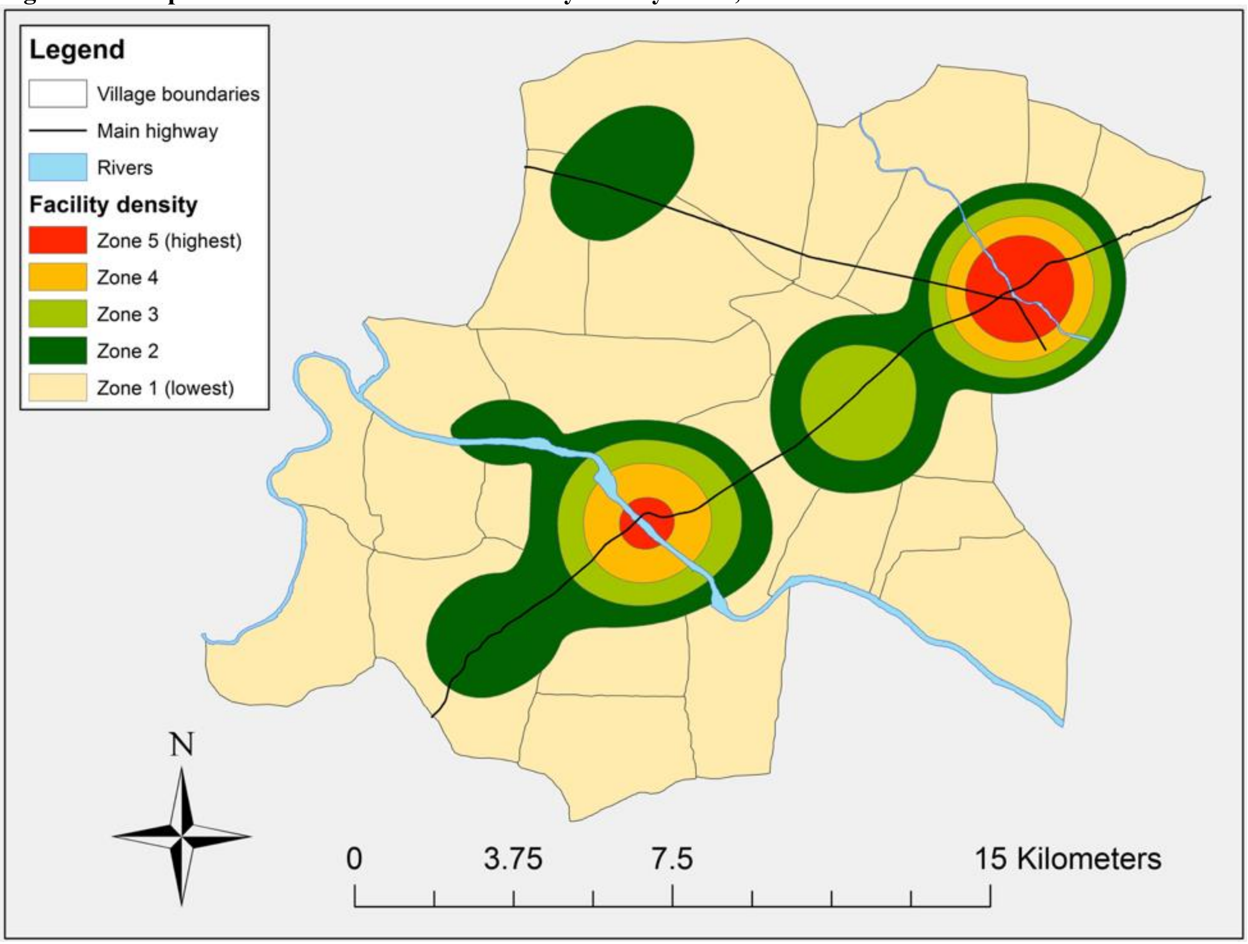

Table A1. Summary GPS visit detection performance when applied to simulated facility visit data, all combinations overall and stratified by parameter values

		Measure of GPS visit detection performance, median (interquartile range)					
	N	Sensitivity	Specificity	AUC	PPV	NPV	Accuracy
All combinations	432	0.93 (0.87, 0.93)	0.92 (0.87, 0.96)	0.91 (0.88, 0.93)	0.64 (0.52, 0.78)	0.99 (0.98, 0.99)	0.92 (0.88, 0.94)
Distance (meters)							
15	54	0.73 (0.60, 0.87)	0.98 (0.98, 0.98)	0.86 (0.79, 0.89)	0.83 (0.80, 0.86)	0.96 (0.94, 0.98)	0.94 (0.91, 0.95)
20	54	0.87 (0.73, 0.93)	0.97 (0.96, 0.97)	0.92 (0.85, 0.95)	0.79 (0.75, 0.81)	0.98 (0.96, 0.99)	0.96 (0.92, 0.96)
25	54	0.93 (0.80, 0.93)	0.96 (0.95, 0.96)	0.94 (0.89, 0.95)	0.77 (0.74, 0.78)	0.99 (0.97, 0.99)	0.95 (0.93, 0.96)
30	54	0.93 (0.93, 0.93)	0.93 (0.91, 0.94)	0.92 (0.91, 0.94)	0.67 (0.61, 0.70)	0.99 (0.99, 0.99)	0.93 (0.91, 0.94)
35	54	0.93 (0.93, 0.93)	0.93 (0.90, 0.93)	0.92 (0.90, 0.93)	0.65 (0.58, 0.67)	0.99 (0.99, 0.99)	0.93 (0.91, 0.93)
40	54	0.93 (0.93, 0.93)	0.92 (0.87, 0.92)	0.91 (0.89, 0.93)	0.62 (0.52, 0.64)	0.99 (0.99, 0.99)	0.91 (0.88, 0.92)
45	54	0.93 (0.93, 1.00)	0.87 (0.82, 0.88)	0.90 (0.87, 0.91)	0.52 (0.45, 0.54)	0.99 (0.99, 1.00)	0.88 (0.85, 0.89)
50	54	0.93 (0.93, 1.00)	0.86 (0.80, 0.87)	0.90 (0.87, 0.90)	0.50 (0.43, 0.52)	0.99 (0.99, 1.00)	0.87 (0.83, 0.88)
Duration (minutes)							
0	72	1.00 (1.00, 1.00)	0.81 (0.75, 0.88)	0.89 (0.87, 0.92)	0.44 (0.37, 0.55)	1.00 (1.00, 1.00)	0.83 (0.78, 0.89)
>1	72	0.93 (0.93, 1.00)	0.91 (0.84, 0.96)	0.92 (0.90, 0.92)	0.60 (0.49, 0.74)	0.99 (0.99, 1.00)	0.91 (0.86, 0.95)
>2	72	0.93 (0.93, 0.93)	0.93 (0.92, 0.97)	0.93 (0.90, 0.94)	0.67 (0.62, 0.78)	0.99 (0.99, 0.99)	0.93 (0.92, 0.95)
>3	72	0.93 (0.87, 0.93)	0.93 (0.92, 0.97)	0.92 (0.90, 0.93)	0.67 (0.61, 0.79)	0.99 (0.98, 0.99)	0.93 (0.91, 0.95)
>4	72	0.93 (0.87, 0.93)	0.93 (0.92, 0.97)	0.92 (0.90, 0.93)	0.67 (0.63, 0.78)	0.99 (0.98, 0.99)	0.93 (0.91, 0.94)
>5	72	0.87 (0.67, 0.87)	0.94 (0.92, 0.97)	0.88 (0.82, 0.90)	0.68 (0.61, 0.77)	0.98 (0.95, 0.98)	0.92 (0.90, 0.94)
Interval (minutes)							
2	48	0.87 (0.60, 0.93)	0.93 (0.90, 0.98)	0.87 (0.77, 0.90)	0.65 (0.57, 0.76)	0.98 (0.94, 0.99)	0.91 (0.89, 0.92)
3	48	0.93 (0.83, 0.93)	0.93 (0.89, 0.96)	0.91 (0.88, 0.92)	0.67 (0.55, 0.75)	0.99 (0.97, 0.99)	0.92 (0.89, 0.93)
4	48	0.93 (0.87, 0.93)	0.93 (0.89, 0.96)	0.92 (0.90, 0.93)	0.67 (0.55, 0.78)	0.99 (0.98, 0.99)	0.93 (0.89, 0.94)
5	48	0.93 (0.87, 0.93)	0.92 (0.87, 0.96)	0.91 (0.89, 0.93)	0.64 (0.52, 0.78)	0.99 (0.98, 0.99)	0.92 (0.88, 0.94)
6	48	0.93 (0.87, 0.93)	0.92 (0.87, 0.96)	0.91 (0.89, 0.93)	0.64 (0.52, 0.78)	0.99 (0.98, 0.99)	0.92 (0.88, 0.94)
7	48	0.93 (0.87, 0.93)	0.92 (0.87, 0.96)	0.91 (0.89, 0.93)	0.64 (0.52, 0.78)	0.99 (0.98, 0.99)	0.92 (0.88, 0.94)
8	48	0.93 (0.87, 0.93)	0.92 (0.87, 0.96)	0.91 (0.89, 0.93)	0.64 (0.52, 0.78)	0.99 (0.98, 0.99)	0.92 (0.88, 0.94)
9	48	0.93 (0.87, 0.93)	0.92 (0.87, 0.96)	0.91 (0.90, 0.93)	0.64 (0.52, 0.78)	0.99 (0.98, 0.99)	0.92 (0.88, 0.94)
10	48	1.00 (0.93, 1.00)	0.91 (0.84, 0.96)	0.92 (0.90, 0.95)	0.60 (0.48, 0.78)	1.00 (0.99, 1.00)	0.91 (0.86, 0.94)

Note: AUC = Area under the receiver operating characteristic curve; PPV = positive predictive value; NPV = negative predictive value

Figure A8. Forest plot of multivariable logistic regression on visit confirmation status by GPS completion threshold used

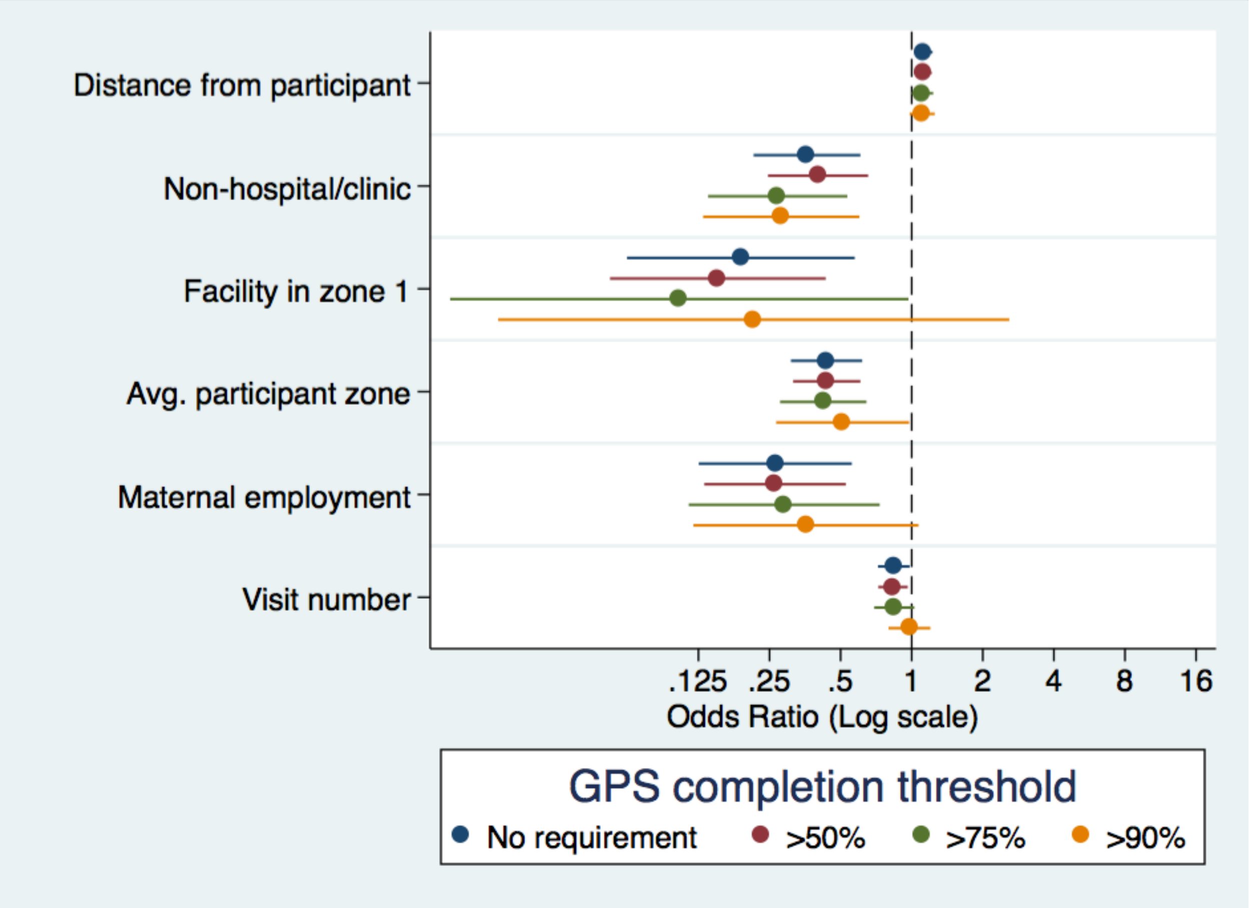

Figure A9. Forest plot of multivariable logistic regression on visit confirmation status by kernel density estimation bandwidth value

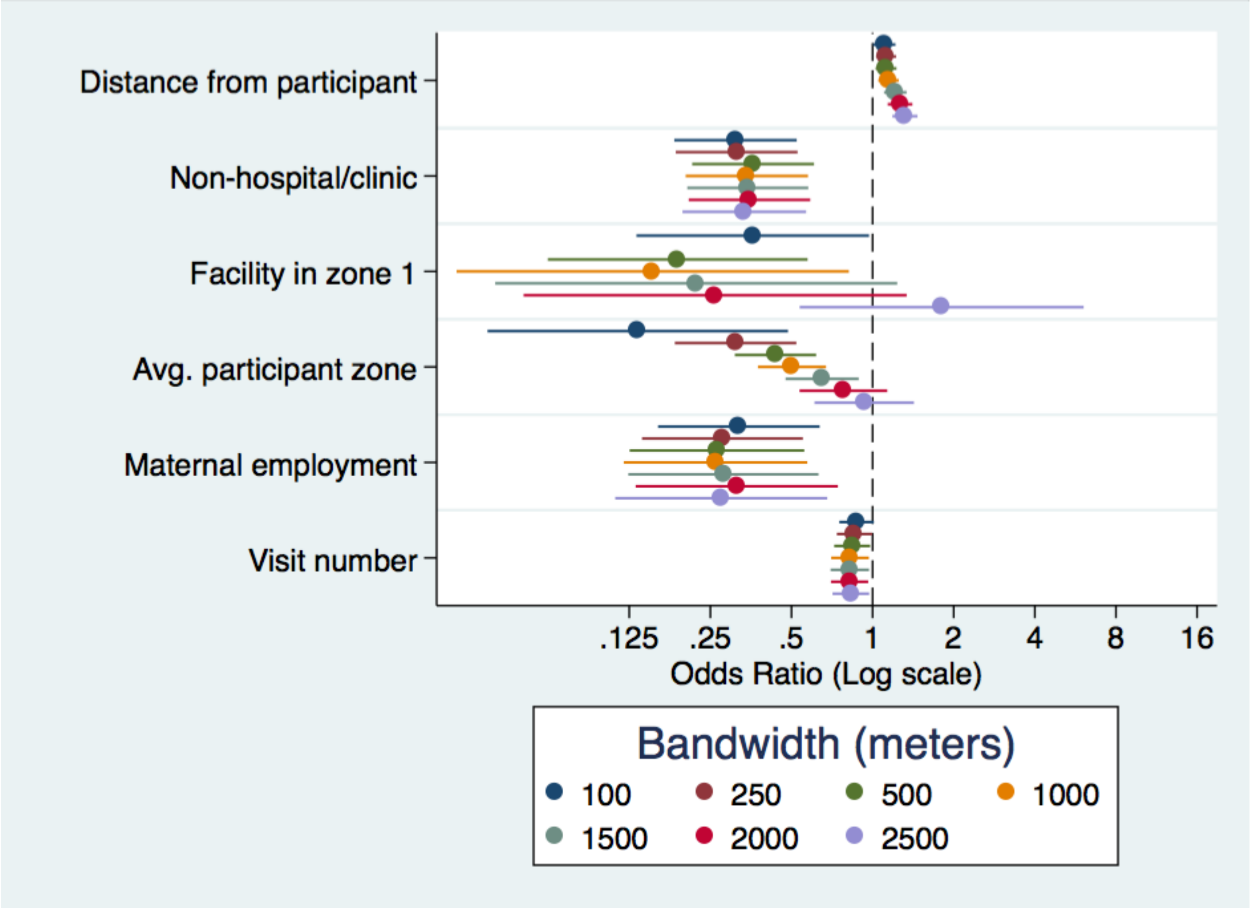

Note: Facility located in zone 1 perfectly predicted failure at a bandwidth to 250 meters and could not be estimated
